# A population-based analysis of clustering identifies a strong genetic contribution to lethal prostate cancer

**DOI:** 10.3389/fgene.2013.00152

**Published:** 2013-08-20

**Authors:** Quentin Nelson, Neeraj Agarwal, Robert Stephenson, Lisa A. Cannon-Albright

**Affiliations:** ^1^Internal Medicine, University of Utah School of MedicineSalt Lake City, UT, USA; ^2^Huntsman Cancer InstituteSalt Lake City, UT, USA; ^3^George E. Wahlen Department of Veterans Affairs Medical CenterSalt Lake City, UT, USA; ^4^Surgery, University of Utah Health Sciences CenterSalt Lake City, UT, USA

**Keywords:** familiality, prostate cancer, lethal, UPDB

## Abstract

**Background:** Prostate cancer is a common and often deadly cancer. Decades of study have yet to identify genes that explain much familial prostate cancer. Traditional linkage analysis of pedigrees has yielded results that are rarely validated. We hypothesize that there are rare segregating variants responsible for high-risk prostate cancer pedigrees, but recognize that within-pedigree heterogeneity is responsible for significant noise that overwhelms signal. Here we introduce a method to identify homogeneous subsets of prostate cancer, based on cancer characteristics, which show the best evidence for an inherited contribution.

**Methods:** We have modified an existing method, the Genealogical Index of Familiality (GIF) used to show evidence for significant familial clustering. The modification allows a test for excess familial clustering of a subset of prostate cancer cases when compared to all prostate cancer cases.

**Results:** Consideration of the familial clustering of eight clinical subsets of prostate cancer cases compared to the expected familial clustering of all prostate cancer cases identified three subsets of prostate cancer cases with evidence for familial clustering significantly in excess of expected. These subsets include prostate cancer cases diagnosed before age 50 years, prostate cancer cases with body mass index (BMI) greater than or equal to 30, and prostate cancer cases for whom prostate cancer contributed to death.

**Conclusions:** This analysis identified several subsets of prostate cancer cases that cluster significantly more than expected when compared to all prostate cancer familial clustering. A focus on high-risk prostate cancer cases or pedigrees with these characteristics will reduce noise and could allow identification of the rare predisposition genes or variants responsible.

## Introduction

Prostate cancer is the most commonly diagnosed cancer in men and is the second leading cause of cancer deaths among men (ACS, 2013). While there is significant evidence of a genetic contribution (Cannon et al., 1982; Carter et al., 1993; Stanford and Ostrander, 2001; Langeberg et al., 2007), decades of investigation into the genetic causes of familial prostate cancer has yet to clearly identify genes or variants which explain much more than a small number of pedigrees with an excess of prostate cancer. Traditional linkage analysis of thousands of high-risk prostate cancer pedigrees has elucidated little in the identification of predisposition genes responsible for prostate cancer pedigrees. This may reflect the heterogeneous nature of prostate cancer, and this could confound identification of informative homogeneous pedigrees segregating rare predisposition variants.

We hypothesize that there exist rare prostate cancer predisposition variants that are responsible for our observation of high risk prostate cancer pedigrees including homogeneous prostate cancer cases (defined by clinical characteristics). We present a methodology to compare subsets of prostate cancer cases and identify those that show more familial clustering than expected for all prostate cancer cases.

Using a population-based resource in Utah that combines genealogy and cancer data, we identified 3 subsets of prostate cancer cases that cluster in pedigrees more than expected: prostate cancer which is diagnosed before age 50 years, lethal prostate cancer (leading to metastasis and death from prostate cancer), and prostate cancer in men with BMI ≥ 30. We propose that analysis of the high-risk prostate cancer cases or pedigrees with an excess of prostate cancer cases with these characteristics could lead to identification of the rare predisposition variants responsible.

## Data and methods

The Utah Population Data Base (UPDB) integrates three key electronic datasets: a Genealogy of the Utah pioneers constructed in the 1970s and kept current (Skolnick, 1980), death certificates for Utah, and a statewide cancer registry. The original Utah genealogy had approximately 1.6 million individual records for 186,000 three-generation families. Since the genealogy was created in the 1970s, state vital records have been used to create genealogy triplets (mother, father, and child) to extend the genealogy to present day. The UPDB has become a person-oriented database with information on 7 million Utahns, some 2.5 million of whom have at least three generations of genealogy. The Utah Cancer Registry (UCR) was created in 1966 to collect data on all cancer diagnosed in Utah. It became a SEER (Surveillance, Epidemiology, and End-Results) Registry of the National Cancer Institute in 1973. The UCR individual records are linked to the Utah genealogy annually; approximately 2/3 of UCR cases link to a record in the UPDB. Cause of death from Utah state death certificates from 1904 to present have been coded to ICD Revisions 6–10, and record linked to the UPDB. Utah Drivers License records from 1970 have been linked to the UPDB and include height and weight measurements for calculation of body mass index (BMI). The combination of genealogy, death certificates, drivers license data, and cancer registry data facilitates the identification of all Utah prostate cancer cases and the genetic relationships between them.

To perform the genetic analyses presented here we restrict ourselves to those individuals in the UPDB with ancestral genealogy data. We identified all individuals in the UPDB who were born before 1972 (when the original Utah genealogy was constructed) and whose parents, four grandparents, and six (of eight total) great grandparents are present in the UPDB genealogy data. This identifies 1.2 million individuals with ancestral genealogy data who are used for all analyses.

We have extended a well-published analysis method, the Genealogical Index of Familiality (GIF), to enable comparison of the relatedness of a subset of prostate cancer cases to the relatedness of *all* prostate cancer cases. Those subsets with evidence for significantly more relatedness than all prostate cancer cases are hypothesized to represent homogeneous genetic subsets that will be most informative for gene identification studies.

### Genealogical index of familiality (GIF) method

For decades the GIF statistic has been used to quantify familial clustering of cancer and other phenotypes in the UPDB. This well-established statistical method has yielded strong evidence of heritability for several cancer phenotypes (Cannon et al., 1982; Cannon-Albright et al., 1994; Larson et al., 2006; Albright et al., 2012). The GIF was developed to test the hypothesis of excess relatedness of individuals with a common phenotype. Excess relatedness is measured by comparing the average relatedness between all pairs of cases of interest to the expected relatedness of matched controls from the Utah population. Since record linkage of any subset of UPDB records may indicate better or different quality data, for individuals with a death certificate, we select controls from all UPDB individuals who have a Utah death certificate. Since the UCR is statewide, we select controls for cancer cases from the entire UPDB resource.

The relatedness of a pair of individuals in a set is measured using the Malécot coefficient of kinship. The Malécot coefficient of kinship mathematically expresses Mendelian inheritance pattern probabilities that randomly selected homologous chromosomes are identical due to inheritance from a common ancestor. For example, the Malécot coefficient for siblings is 1/4, avunculars is 1/8, and first cousins 1/16. The GIF analysis tests excess relatedness by comparing all pairwise relationships within a set of cases to the expected relatedness measured in all pairwise relationships in 1000 sets of matched controls randomly selected from the UPDB. Controls were matched on characteristics that might be associated with record linking and disease rates, including five-year birth year cohort, sex, and birth state (Utah or not).

The overall GIF analysis tests for significant excess relatedness (over what is expected in the UPDB population) among a group of individuals. It can be performed on all prostate cancer cases, and on subsets of cases based on cancer characteristics. It cannot, however, determine which, if any, of these subsets exhibits the best evidence for a genetic predisposition, and which therefore might be the best set of high-risk pedigrees in which to search for genes.

### New SubsetGif test

Here we consider a modified GIF test and test the relatedness of multiple subsets of prostate cancer cases to identify those which exhibit excess relatedness above the observed relatedness among *all Utah prostate cancer cases*. This modified GIF test is referred to as the SubsetGif. Evidence for significant excess relatedness for a subset of prostate cancer cases above the expected for *all prostate cancer cases* could indicate the presence of a common genetic cause shared by the homogeneous subset. The identification and subsequent study of pedigrees including cases of such a homogeneous subset might facilitate the identification of rare predisposition genes.

### Contribution to the GIF by genetic distance

It is possible to view the distribution of the contribution to the GIF statistic by the pairwise genetic distance of the different relationships observed in cases (and controls). The genetic distance represents the number of paths between a pair of individuals. Genetic distance 1 represents parent/offspring pairs, genetic distance 2 represents siblings or grandparent/grandchild, genetic distance 3 represents avunculars, and so forth.

## Results

In the UPDB resource, 18,291 prostate cancer cases were identified who also had ancestral genealogical records. The available prostate cancer subsets and their corresponding sample sizes are outlined in Table 1.

**Table 1:** **Subsets of prostate cancer and sample size**.

**Set of prostate cancer cases**	***n***
All prostate cancers	18,291
Age at diagnosis <50 years	213
Metastatic disease at diagnosis	912
With at least 1 primary cancer of other site	2922
Gleason score >7 at diagnosis	4784
Short survival (0–9 months)	1180
Long survival (240 + months)	806
High BMI (≥30)	2459
Prostate cancer cause of death (lethal prostate cancer)	3982

### Analysis of excess relatedness

Previous studies have strongly supported evidence for a genetic contribution to predisposition to prostate cancer in the Utah population, as well as other populations (Cannon et al., 1982; Cannon-Albright et al., 1994, 2005). When all prostate cancer cases with genealogy data in the UPDB are analyzed there is evidence of excess relatedness (represented by both close and distant genetic relationships) over expected relatedness in matched Utah population controls. Table 2 shows the traditional GIF test for excess relatedness compared to matched Utah population controls for all prostate cancer cases, and for each subset. The mean relatedness for cases and controls is shown. All prostate cancer cases and subsets, except prostate cases who survived less than 10 months after diagnosis, show strong evidence for excess clustering compared to Utah population controls. These results suggest a genetic contribution to prostate cancer predisposition, and suggest that study of almost all subsets of prostate cancer could be fruitful, but the results do not allow identification of which, if any, of the subsets are significantly more related than expected when compared to all prostate cancer cases, and thus show the best evidence for a genetic contribution.

**Table 2:** **GIF analysis of prostate cancer relatedness compared to expected relatedness in the UPDB population**.

**Group**	***n***	**Case GIF**	**Mean control GIF**	**Empirical significance**
All prostate cancers	18,291	5.54	4.74	<0.001
Age at diagnosis <50 years	213	11.72	4.54	<0.001
Metastatic disease at diagnosis	912	5.94	4.89	<0.001
With at least 1 primary cancer of other site	2922	5.58	4.74	<0.001
Gleason score >7 at diagnosis	4784	5.41	4.69	<0.001
Short survival (0–9 months)	1180	5.19	4.92	0.138
Long survival (240 + months)	806	5.64	4.75	0.005
BMI ≥ 30	2459	5.81	4.71	<0.001
Prostate cancer cause of death^*^ (lethal)	3982	5.98	4.93	<0.001

* *Because the subset of lethal prostate cancer cases differs from all prostate cancer cases with respect to the identification of a linked death certificate record, and because the fact of record linking may suggest different data quality, we performed the GIF analysis for the subset of cases with prostate cancer contributing to death in Tables 2, 3 using only the 10,421 prostate cancer cases with a linked Utah death certificate as controls; this is the standard for analysis of sets of individuals selected from Utah death certificate data (Cannon-Albright, 2008)*.

In order to consider the hypothesis that a subset of prostate cancer cases represents a more homogeneous subset of highly related cases, we propose use of the SubsetGif analysis. The average pairwise relatedness of each subset of cases is compared to the average pairwise relatedness of 1000 sets of matched “controls”; these controls are selected from the set of 18,291 Utah prostate cancer cases. The results for this SubsetGif test are shown in Table 3. The average pairwise relatedness of the cases does not change for any subset (as expected), but the mean control GIF statistic is higher than in Table 2 for each subset because the “controls” here are randomly selected prostate cancer cases, who are more closely related than random members of the Utah population.

**Table 3:** **Subset prostate cancer relatedness compared to expected prostate cancer case relatedness in the UPDB**.

**Prostate cancer subsets**	***n***	**Case GIF**	**Mean control GIF**	**Empirical significance**
Age at diagnosis <50 years	213	11.72	7.51	0.024
Metastatic disease at diagnosis	912	5.94	5.95	0.506
With at least 1 primary cancer of other site	2922	5.58	5.51	0.303
Gleason Score >7 at diagnosis	4784	5.41	5.39	0.417
Short Survival (0–9 months)	1180	5.19	6.08	1.000
Long Survival (240 + months)	806	5.64	5.56	0.400
BMI ≥ 30	2459	5.81	5.27	<0.001
Prostate cancer cause of death (lethal)	3982	5.98	5.76	0.030

Controls randomly selected from 18,291 prostate cancer cases.

Table 3 results show that the average pairwise relatedness of three different subsets of prostate cancer cases is significantly higher than expected among prostate cancer cases, supporting the hypothesis that these subsets of cases cluster more than all prostate cancer cases and represent sets on which to focus for predisposition gene identification. The three subsets include prostate cancer cases diagnosed before age 50 years, prostate cancer cases with BMI ≥ 30, and prostate cancer cases whose cause of death is prostate cancer (lethal prostate cancer).

It is difficult to determine whether these three subsets represent independent groups of interest or whether there is overlap between the groups because not all cases have BMI and death certificate data. There were 222 prostate cancer cases with BMI ≥ 30 among the 3982 cases with prostate cancer as a cause of death (6% total and 17% of the 1300 lethal cases with BMI data), and 58 prostate cancer cases with BMI ≥ 30 of the 213 cases who were diagnosed before age 50 years (27%). Overall, 11,536 prostate cancer cases had BMI data, and 21.3% were BMI ≥ 30. There were 26 prostate cancer cases diagnosed before age 50 years (0.7%) among the 3982 lethal prostate cancer cases, and overall the 213 prostate cancer cases diagnosed before age 50 years represented 1% of all cases.

**Figure 1:**
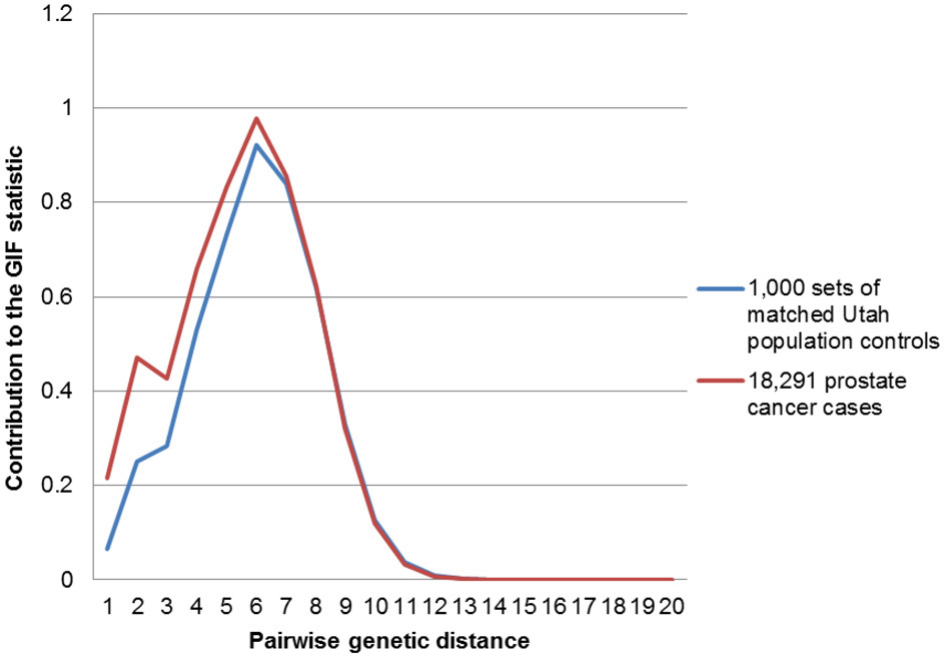
**Contribution to the GIF statistic by pairwise genetic distance for cases and controls for all prostate cancers vs. population**.

In order to determine the overall distribution of excess relatedness we can view the contribution to the GIF statistic by the pairwise genetic distance for cases and for controls. Figure 1 shows the GIF distribution for all 18,291 prostate cancer cases compared to the distribution for the 1000 sets of matched Utah population controls. The comparison shows that the relatedness for prostate cancer cases exceeds that expected in the Utah population, as observed in random matched Utah controls, for genetic distances up to 7 (e.g., second cousins once removed).

**Figure 2:**
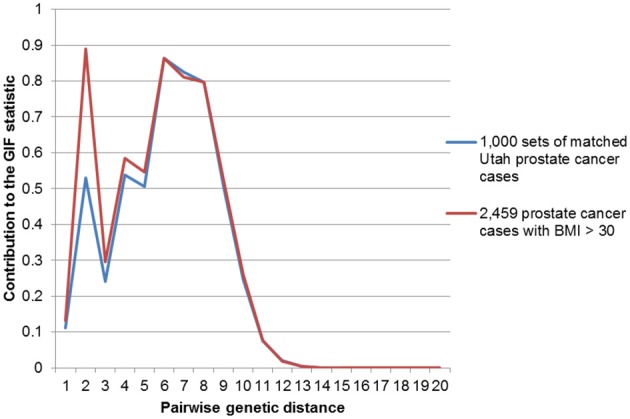
**Contribution to the GIF statistic by pairwise genetic distance for cases and controls for prostate cancer cases with a BMI of 30 or greater**.

**Figure 3:**
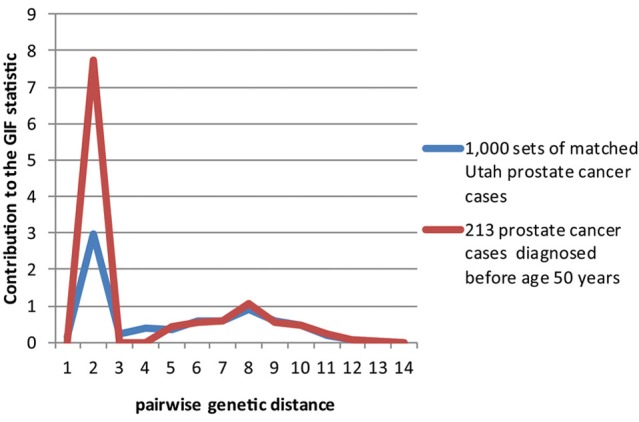
**Contribution to the GIF statistic by pairwise genetic distance for cases and controls for prostate cancer cases diagnosed before age 50**.

**Figure 4:**
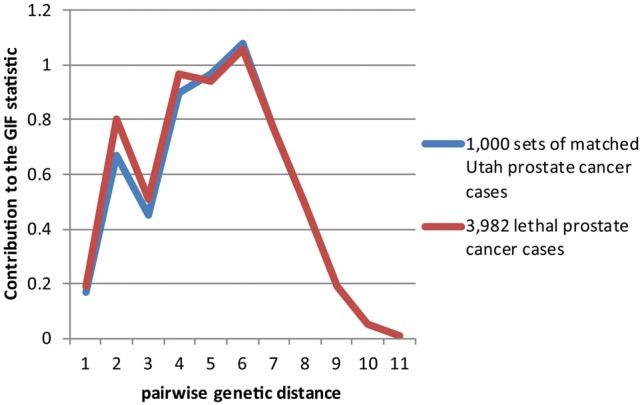
**Contribution to the GIF statistic by pairwise genetic distance for cases and controls for prostate cancer cases that have prostate cancer as a cause of death**.

Figures 2–4 show the contribution to the GIF statistic for the three subsets of cases, with matched controls randomly selected from all Utah prostate cancer cases. Figure 2 shows this distribution for prostate cancer cases with BMI ≥ 30; as seen in Table 3 there is significant excess relatedness for prostate cases with BMI ≥ 30. This excess extends to a genetic distance of 5, equivalent to first cousins once removed, for example. Figure 3 shows this distribution for prostate cancer cases diagnosed before age 50 years, which is also observed to show significant excess relatedness. The excess relatedness is irregular, but is clearly observed for genetic distance = 2 (siblings primarily), and distance = 8 (third cousins, for example). Figure 4 shows the GIF distribution for lethal prostate cancer cases, also observed to show significant excess clustering when compared to all deceased prostate cancer cases. The excess extends to genetic distance = 4, equivalent to first cousins, for example.

**Figure 5:**
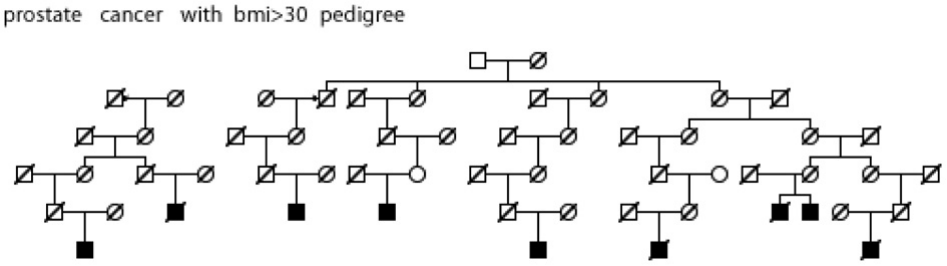
**High risk Utah prostate cancer pedigree (56 prostate cancer cases observed among descendants of the pedigree founder, 36 expected, *p* = 0.001); cases with BMI ≥ 30 are shown**.

**Figure 6:**
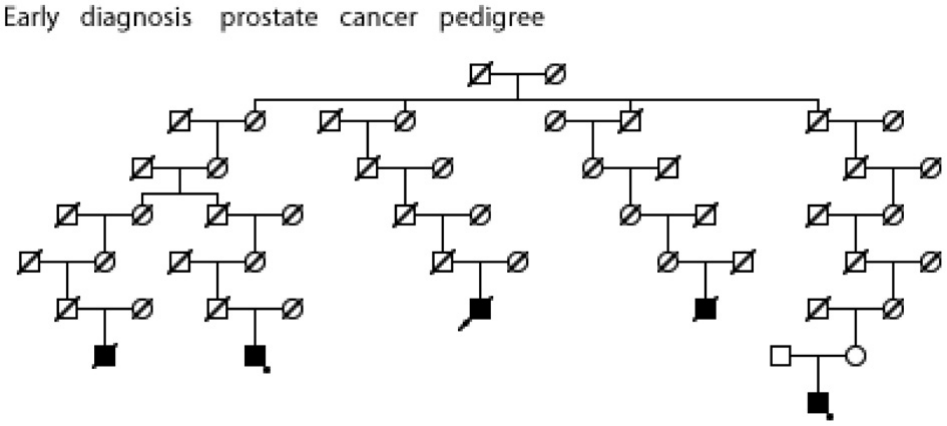
**High risk Utah prostate cancer pedigree (173 prostate cancers observed among descendants of the pedigree founder, 131 expected, *p* = 0.0003); cases diagnosed before age 50 years are shown.** The two cases with an asterisk were also observed to have BMI ≥ 30 (data not available for all cases).

**Figure 7:**
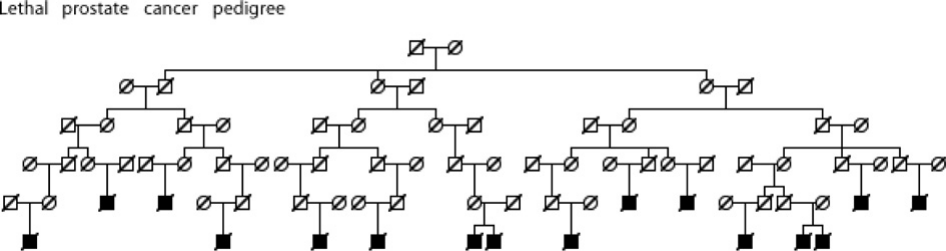
**High risk Utah prostate cancer pedigree (76 prostate cancer cases observed among descendants of the pedigree founder, 51.5 expected, *p* = 0.0008); cases known to have died from prostate cancer are shown**.

Figures 5–7 show examples Utah high-risk prostate cancer pedigrees for each of the subset characteristics identified.

## Discussion

Analysis of a population-based Utah resource linking cancer characteristics data with genealogy data has previously shown evidence for a genetic contribution to prostate cancer predisposition (Cannon et al., 1982; Cannon-Albright et al., 1994, 2005; Albright et al., 2012; Teerlink et al., 2012). Here we have extended a well-published analysis method which tests for excess relatedness in a set of individuals to allow the identification of subsets of prostate cancer cases who show the strongest evidence for excess familial clustering. The subsets identified might be argued to represent the most informative sets of cases or pedigrees to be studied for rare predisposition gene identification.

Some of the subsets of prostate cancer cases that show significant evidence of clustering in excess of expected for prostate cancer were expected, some represent new subsets of interest for genetic studies. The subset of men diagnosed with prostate cancer before age 50 years is not surprising; there is much literature suggesting a strong genetic contribution to cancer of most sites that is diagnosed early (Goldgar et al., 1994; Brandt et al., 2008) and much analysis of this subset of prostate cancer cases and pedigrees has been performed (Gronberg et al., 1999; Xu et al., 2005). However, the other two groups of prostate cancer cases identified, high BMI (≥30) and lethal prostate cancer cases, have not been suggested previously as associated with a strong genetic contribution for prostate cancer. There was some overlap of prostate cancer cases between these sets; further investigation of specific high-risk pedigrees will determine whether they are independent.

Although epidemiologic studies have shown that systemic metabolic disorders including obesity might increase risk for prostate cancer, BMI in the context of high risk prostate cancer pedigrees does not appear to have been studied. Since there is evidence for familial clustering of high BMI or obesity (independent of cancer status), it is possible that these results are due, at least in part, to a shared predisposition to obesity. Nevertheless, these results suggest this is an informative set of pedigrees to be studied for prostate cancer risk.

The familiality of *aggressive* prostate cancer has been noted, and subsets of aggressive prostate cancer cases have been studied, without any gene identifications (Paiss et al., 2003; Lange et al., 2006; Schaid et al., 2006; Christensen et al., 2007). Little progress has been made in understanding why 30% of all patients with localized prostate cancer eventually develop recurrent, and subsequently fatal, prostate cancer. Rather than subset aggressive prostate cancers, we specifically targeted the pathogenesis of lethal prostate cancer. This subtle definition difference focuses on the subtype of prostate cancer which is associated with the worst prognosis i.e., which kills, but our definition ignores age at onset and pathology grading data for the individual, both of which are more commonly used to classify prostate cancer cases for aggressive status, but which can be poor markers for survival. This subset of lethal prostate cancer cases, among all others, is the most clinically significant and that which could yield the most translational opportunities were genes to be identified.

The Utah population has proven valuable to the study of many common cancers, and to the isolation of multiple cancer predisposition genes. The University of Utah group has been studying high-risk cancer pedigrees since 1972, and has built a resource of thousands of extended high-risk pedigrees that includes over 35,000 DNA samples. The study of extended pedigrees allowed our research group to isolate *BRCA1* (Miki et al., 1994), to localize and isolate *BRCA2* (Wooster et al., 1994; Tavtigian et al., 1996), to localize and isolate *p16* (Cannon-Albright et al., 1992, 1994; Kamb et al., 1994), and to localize and isolate *HPC2/ELAC2* (Tavtigian et al., 2001). These findings of excess relatedness in the UPDB for three subsets of prostate cancer cases represent multiple Utah high-risk prostate cancer pedigrees for each of the subsets. Analysis of these high risk pedigrees will lead to identification of the predisposition genes responsible, which might otherwise not be identifiable in studies of all high-risk prostate cancer pedigrees combined.

We have identified significant evidence for three characteristics of prostate cancer that independently coaggregate in both close and distant relatives. We have identified multiple high-risk prostate cancer pedigrees that independently include multiple prostate cancer cases with the characteristics of interest. Figures 5–7 show an example Utah high-risk prostate cancer pedigree for each of the three characteristics identified. We propose that linkage analysis or shared genomic segment (Thomas et al., 2008) analysis can identify chromosomal regions shared in the related cases and that sequence analysis of predisposition carriers in the targeted regions located will lead to identification of the responsible predisposition genes. Rather than studying all high-risk prostate cancer pedigrees, we instead will focus on those that exhibit multiple cases with those characteristics most likely to have a genetic contribution. These studies will examine fewer pedigrees than a typical prostate cancer pedigree study, but will focus on the homogeneous subsets most likely to represent rare segregating predisposition genes or variants.

These findings should be generalizable to the U.S.A. population. Utah was originally settled by ~10,000 Mormons of British, Scandinavian, and German origin. They, and the more than 50,000 migrants from the same areas who arrived in the next generations, have typical Northern European gene frequencies (McLellan et al., 1984) and low to normal levels of inbreeding compared to the U.S. (Jorde, 1989). These characteristics make this population appropriate for inferences in populations of Northern European descent. The predisposition genes identified in Utah are represented similarly in other studies in terms of frequency, penetrance, and interactions with risk factors and modifier genes. Utah cancer rates are lower than U.S. rates, most likely due to lower rates of smoking and alcohol use.

Recent advances in mapping the genome, combined with the unique resources of Utah, provide a rare opportunity for a successful search for predisposition genes or variants for prostate cancer and the definition of their role at a population level. Recent evidence has shown the advisability and efficiency of rare predisposition gene identification by study of extended pedigrees (Ewing et al., 2012; Roberts et al., 2012). Here we identify characteristics of prostate cancer that can be used to more specifically focus gene identification efforts on appropriate pedigrees. The eventual identification of predisposition genes for prostate cancer, accompanied by a greater understanding of how these genes contribute to morbidity and mortality, will lead to the development of diagnostic tests and more personalized treatments for prostate cancer.

### Conflict of interest statement

The authors declare that the research was conducted in the absence of any commercial or financial relationships that could be construed as a potential conflict of interest.
